# Does forced whisper have an impact on voice parameters?

**DOI:** 10.1007/s00405-024-08698-7

**Published:** 2024-05-06

**Authors:** Matthias Echternach, Marie Köberlein, Michael Döllinger, Jonas Kirsch, Theresa Pilsl

**Affiliations:** 1 Division Phoniatrics and Pediatric Audiology, Department of Otolaryngology, Munich University Hospital and Faculty of Medicine, Munich University (LMU), Campus Großhadern, Marchioninistraße 15, 81377 Munich, Germany; 2 Division of Phoniatrics and Pediatric Audiology, Department of Otolaryngology Head & Neck Surgery, University Hospital Erlangen, FAU Erlangen-Nuremberg, Erlangen, Germany

**Keywords:** Whispering, Loading, Voice production

## Abstract

**Objectives:**

There has been the assumption that whispering may impact vocal function, leading to the widespread recommendation against its practice after phonosurgery. However, the extent to which whispering affects vocal function and vocal fold oscillation patterns remains unclear.

**Methods:**

10 vocally healthy subjects (5 male, 5 female) were instructed to forcefully whisper a standardized text for 10 min at a sound level of 70 dB(A), measured at a microphone distance of 30 cm to the mouth. Prior to and following the whisper loading, the dysphonia severity index was assessed. Simultaneously, recordings of high speed videolaryngoscopy (HSV), electroglottography, and audio signals during sustained phonation on the vowel /i/ (250 Hz for females and 125 Hz for males) were analyzed after segmentation of the HSV material.

**Results:**

The pre-post analysis revealed only minor changes after the intervention. These changes included a rise in minimum intensity, an increase in the glottal area waveform-derived open quotient, and the glottal gap index. However, no statistically significant changes were observed in the harmonic-to-noise-ratio, the glottal- to-noise-excitation-ratio, and the electroglottographic open quotient.

**Conclusion:**

Overall, the study suggests that there are only small effects on vocal function in consequence of a forced whisper loading.

## Introduction

The voice plays a crucial role in human communication, and any impairment can lead to dysphonia resulting in the consequence of communication disabilities [1]. Dysphonia can be characterized by the deterioration of voice quality, such as hoarseness, limitations of fundamental frequency (*ƒ*_o_) range, sound pressure level (SPL), endurance, and other symptoms such as coughing, dysphagia, breathing problems, etc. [2].

Vocal loading, particularly, increases both, vocal impact and shear stress on the vocal fold tissue, potentially leading to inflammation [3]. Previous studies have demonstrated that vocal loading, as measured by an increase of vocal dose – calculated through various accelerometer-based definitions [4–11] – could influence vocal function. Additionally, for voice professionals, such as teachers, priests, actors, singers or call-center-employees, vocal loading may not only influence personal communication but also result in economic challenges [12].

However, assessing the impact of vocal loading on vocal function in clinical settings is a challenging task. To measure such effects, various vocal loading tests have previously been established, evaluating vocal function during or after a defined vocal loading task. These tests typically involve the patient phonating at a minimum sound pressure level (SPL) for a specific duration. Nevertheless, as stated in a previous investigation [13], these tests exhibit variations in terms of time intervals (10 min [8, 13–16], 16 min [17] up to hours [18] or repetitions such as 3 × 15 min[3] or 5 × 45 min [19]), the minimal sound pressure level (from 65 dB, 80 dB [13, 14, 19, 20] to 90 dB [3] or reading against an ambient noise [21]), the distance to the sound level meter (from 2 m [19], 50 cm [22], 40 cm [23] or 30 cm [8, 13, 14, 17], the dB weighting (A [8, 13] or C [20]), the type of vocalization (standardized text [15, 22], reading a text of the subject’s choice [19], counting numbers [24], vocalization of vowels [17, 20]), sitting or standing position [19], and whether the minimal SPL changed in intervals during the test [15, 17, 22] or not [13, 14, 20]. Previous studies have shown that vocal fold oscillations were influenced after vocal loading tests, as recorded using stroboscopy [18, 25], furthermore concerning the Phonation Threshold Pressure [26, 27], the self-estimation of vocal function using the Voice Handicap Index (VHI [28]) [15], acoustic measures [15, 29, 30], or the Dysphonia Severity Index (DSI [31]) [8, 14, 32].

Inflammatory reactions due to vocal loading are feared especially after phonomicrosurgery. The wound-healing process begins immediately after surgery, and can take anywhere from several weeks to several months [33], depending on the extent and depth of damage [34], as well as the components necessary for healing, such as protein and proteoglycan synthesis, and the element of wound contraction. Concerning this phase, there is ongoing debate about whether there should be voice rest, or a relaxed, soft, and low voice use, or unrestricted speaking voice [34, 35]. Furthermore, the optimal duration for applying such practices is still under discussion [36, 37].

To avoid voice production, many patients use whispering for communication. During whispering, there is no vocal fold oscillation [38] and no vocal fold closure, with the consequence of the development of turbulences producing noise as a sound source – an aero-acoustic sound production. The subglottic pressure is considered not high [39]. Furthermore, it has been shown that there are different glottal and supraglottal configurations associated with whispering [40]. Although one might anticipate minimal stress on the vocal folds due to the absence of oscillation and low subglottic pressure, it has been frequently suggested that such aero-acoustic voice production during whispering could lead to malregulation and vocal hypertension [41]. Furthermore, some authors differentiate between a relaxed and un-tensioned whispering compared to forced and tensioned whispering [38]. While some authors recommend avoiding whispering altogether, others permit untensioned and relaxed whispering after surgery [38]. However, the detailed impact of whispering on vocal function, especially regarding forced, tensioned whispering, has not yet been fully understood.

The presented study aims to examine alterations in vocal fold oscillation and vocal function following a standardized forced whisper loading test in vocally healthy subjects, employing high speed digital videolaryngoscopy (HSV), audio and electroglottographic (EGG) signals. It was hypothesized that vocal function would be reduced subsequent to the whisper loading.

## Materials and methods

After approval of the local ethical committee, ten vocally untrained subjects (5 female, 5 male, age 25–49 years) participated in this study after giving their informed written consent. None of the subjects had a medical history of vocal dysfunction or acute voice complaints.

All subjects were asked to perform a standardized whisper loading test, analogous to a standardized vocal loading test outlined in previous studies [13, 14, 32]. Here, the subjects were required to engage in forced, tensioned whispering of a predefined, standardized text (Grimm Brothers: Das tapfere Schneiderlein) for a duration of 10 min while maintaining a SPL higher than 70 dB(A), measured at a distance of 30 cm from the mouth. Analogous to the recommendations of the German society of Phoniatrics and Pediatric Audiologists for standardized vocal loading tests, the whisper loading test was conducted in an acoustically untreated environment, simulating a quasi-living-room acoustic setting. The LingWaves software (Wevosys, Forcheim, Germany) facilitated the test, signaling on the computer screen when the SPL would fall below the required 70 dB(A). The SPL (dB(A)) and the deviation of the 70 dB criterion were then calculated as means for each minute over the 10-min duration of the performance.

In line with previous investigations [13], the DSI (Wevosys, Forchheim, Germany with the sound level meter Tecpel 331, Taipei, Taiwan) was calculated both before and immediately after the whisper loading test. The DSI computation included measurements of minimum intensity and highest *ƒ*_o_ – both derived from the voice range profile function of the Lingwaves software –, the maximum phonation time (best of 3 attempts, vowel /a/, comfortable pitch and loudness), and an audio signal recording during sustained phonation on the vowel /a/ at comfortable pitch for the determination of the jitter.

Immediately before and after the whisper loading test, a flexible transnasal video endoscopy (HSV, Fastcam SA-X2 (Photron, Tokyo, Japan) using a flexible endoscope (ENF GP; Fa. Olympus, Hamburg, Germany), 300W light source (Storz, Tuttlingen, Germany) with a spatial resolution of 386 × 320 pixels at 20.000 frames per second with simultaneous recording of the electroglottography (EGG, EG2-PCX2, Glottal Enterprises (Syracuse, NY)) and audio signals (DPA d:screet 4061 core (DPA microphones, Alleroed, Denmark)) was performed, as described elsewhere [42–44]. During the recording, the participants were instructed to sustain phonation on the vowel /i/ at 250 Hz for female and 125 Hz for male voices, respectively, starting with higher loudness and gradually decreasing loudness during sustained phonation. For the pre-post comparison, the analyzed interval extended from 200 ms after the voice onset to 200 ms before the voice offset. However, for four subjects, this interval had to be a bit shortened due to artifacts at the recording`s end in the EGG signal. The audio signal was calibrated using the Sopran software (Svante Granqvist, Karolinska Institute, Stockholm, Sweden) with a reference sound meter recording.

To analyze the HSV footage, the glottis was segmented utilizing the Glottis Analysis Tools Software [45]. Phonovibrograms were then generated from the segmented glottis [46, 47]. Subsequently, the Glottal Area Waveform (GAW) along with the corresponding audio and EGG signal, were analyzed using the Multi Signal Analyzer (Division of Phoniatrics, University Hospital Erlangen, Germany). This software facilitated the calculation of numerous numerical data analogous to the Glottal Analysis Tools across different signal types. The variables specific to this study are presented in Table 1. For the estimation of the EGG open quotient, the Howard criterion [48] was applied.

**Table 1:** Computed parameters for the three signal types (Audio, electroglottography (EGG), Glottal Area Waveform (GAW)). Parameters were computed based on the formulas provided in [50]

Audio	EGG	GAW
Amplitude perturbation quotient	Amplitude perturbation quotient	Amplitude perturbation quotient
Cepstral peak prominence	Cepstral peak prominence	Cepstral peak prominence
Dysphonia severity index	Fundamental frequency	Closing quotient
Glottal to noise excitation ratio	Glottal to noise excitation ratio	Glottal to noise excitation ratio
Harmonic to noise ratio	Harmonic to noise ratio	Glottal gap index
Relative average perturbation	Open quotient	Open quotient
Sound pressure level_mean_	Relative average perturbation	Relative average perturbation

Wilcoxon Signed-Rank tests were employed to analyze pre-post differences (JAPS, version 0.18.3, University of Amsterdam, The Netherlands), with the level of significance set at p ≤ 0.05 and statistical tendency was set at p ≤ 0.10.

## Results

**Fig. 1:**
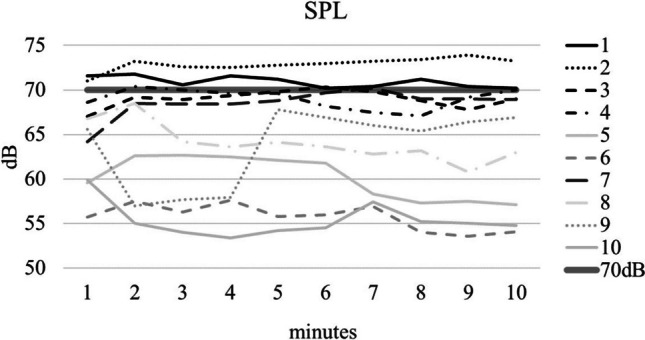
Mean Sound Pressure Levels for each minute of the whisper loading test for 10 subjects. The 70 dB criterion line is marked in bold grey. Black graphs represent the TW group (tensioned whisperers), while grey graphs represent the UW group (untensioned whisperers), see text

All subjects completed the whisper loading test without interruption. However, as shown in Fig. 1, there was a large variance around the 70 dB criterion.

The extent of under-fulfilment ranged from 0.4% to 100% of the total 10-min duration. Considering that this criterion may be indicative of a more or less forceful tensioned whispering, the median dB(A) was measured at 67.1 dB(A) (range 53.4–73.9 dB). For a detailed analysis, the 5 subjects with higher dB values were categorized as tensioned whisperers (TW, represented by black lines in Fig. 1), while the 5 subjects with lower values were categorized as untensioned whisperers (UW, represented by grey lines in Fig. 1), respectively.

In the pre-post comparison, the DSI exhibited no statistical difference. However, as indicated in Table 2, the minimum intensity – in contrast to all other components defining the DSI – showed a statistically significant increase after the intervention.

**Table 2:** Pre-post comparison with mean values and standard deviation (SD) for the Dysphonia Severity Index (DSI), minimum intensity (I_min_), maximum phonation time (MPT), jitter and maximum fundamental frequency (F_max_)

	Pre		Post		p value
	Mean	SD	Mean	SD	
**DSI**	7.84	1.84	7.50	2.48	0.407
I_min_ (dB)	46.87	3.34	49.28	5.62	**0.049**
MPT (s)	26.39	7.33	27.02	8.46	0.922
Jitter (%)	0.18	0.10	0.23	0.18	0.678
F_max_ (Hz)	841.10	234.50	879.00	278.15	0.343

The p values refer to the Wilcoxon Signed-Rank Test. Bold p-value refers to a stastistically significance below 0.05

**Fig. 2:**
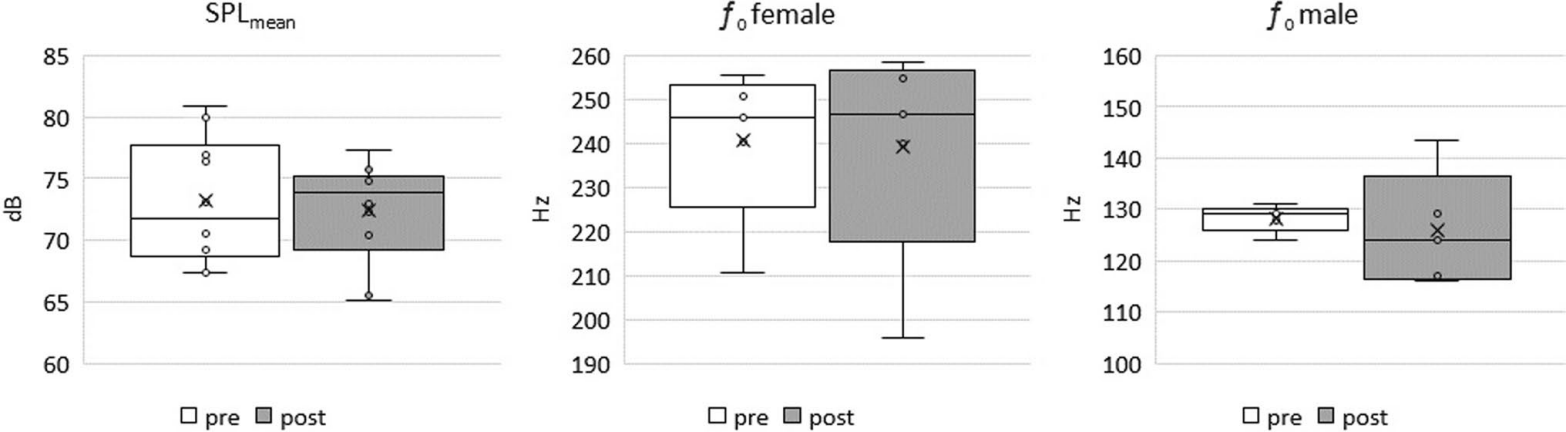
Mean Sound Pressure Level (SPL) and fundamental frequency (*ƒ*_o_, from the EGG signal) for female and male subjects

**Fig. 3:**
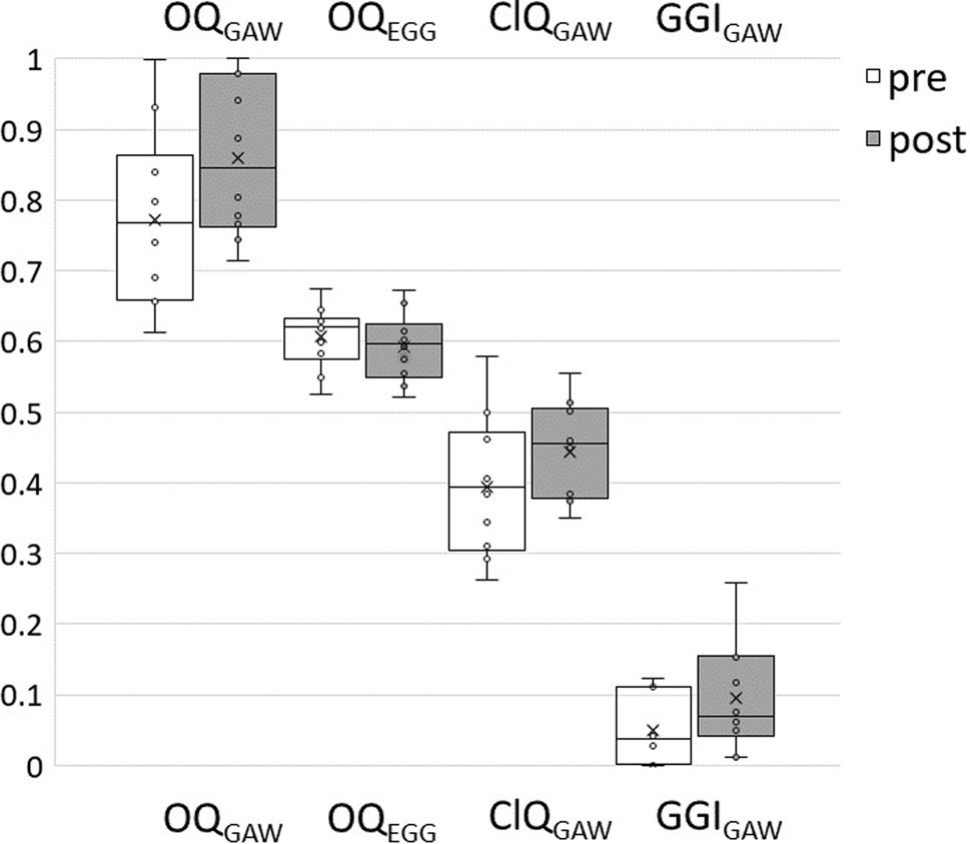
Glottal area waveform (GAW) derived and electroglottographic (EGG) derived Open quotients (OQ_GAW_ versus OQ_EGG_, respectively), Closing Quotient (ClQ) and Glottal Gap Index (GGI) for the pre and post intervention measurements

During sustained phonation, there were no statistically significant changes in SPL for the given *ƒ*_o_, as illustrated in Fig. 2. Across all subjects there was a statistical tendency towards an increase in OQ_GAW_. (p = 0.10), see Fig. 3. For the TW group all subjects showed such an increase whereas for the UW group only 3 out of 5 showed such a rise. Furthermore, a statistically significant increase was observed for the GGI with a p value of 0.029. For all subjects of the TW group there was an increase of GGI and the median pre to post difference was 0.034 vs. 0.012 for the UW group.

**Fig. 4:**
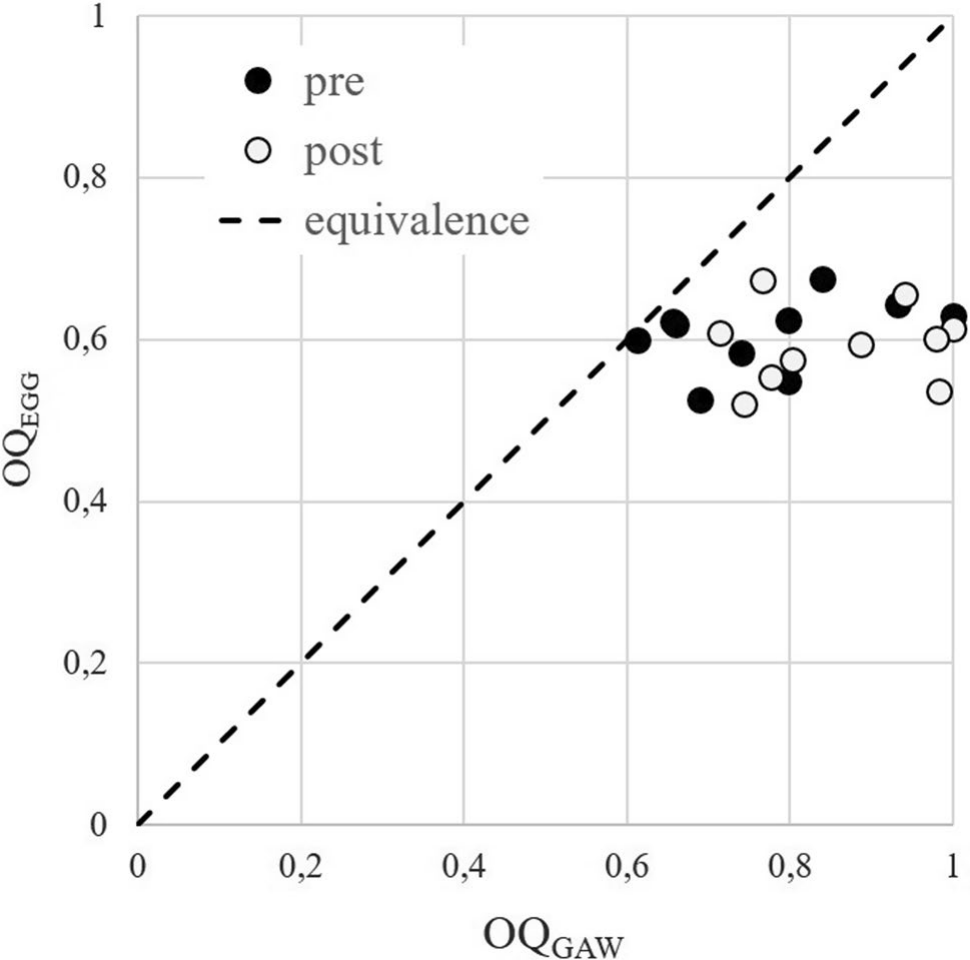
OQ_GAW_ versus OQ_EGG_ for the pre (black) and post (white) measurements. The dotted line refers to 100% concordance

**Fig. 5:**
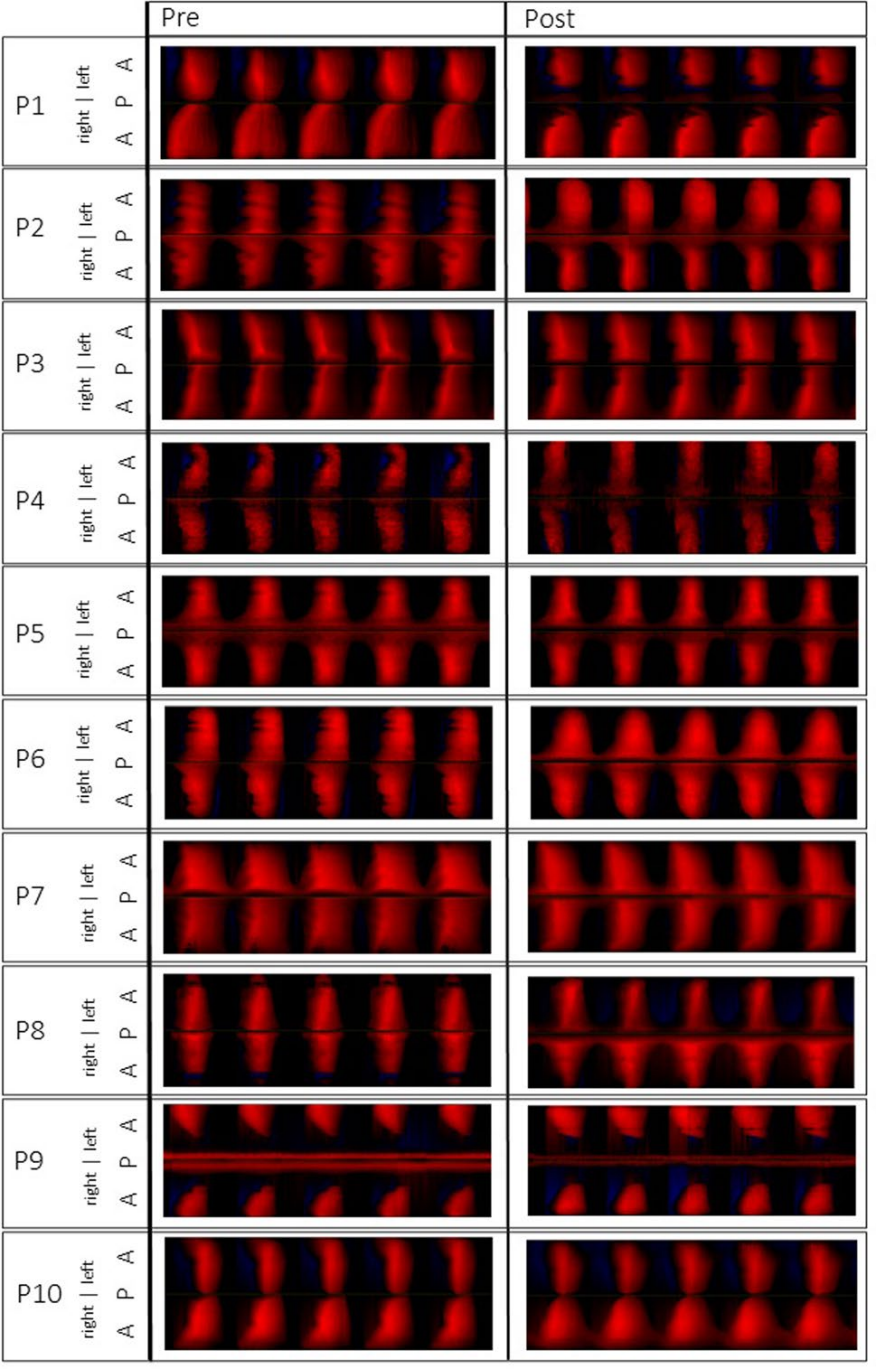
Phonovibrograms representing pre and post recordings for all subjects. The intensity of the red colour corresponds to the distance from the glottis midline. *A* anterior, *P* posterior

OQ_EGG_ and ClQ differed not statistically significant after the whisper loading test. Figure 4 shows the OQ_GAW_ versus OQ_EGG_. As shown in the phonovibrograms, the rise of OQ_GAW_ was neither associated with changes of phase asymmetries or major disturbance of periodicity, see Fig. 5.

**Fig. 6:**
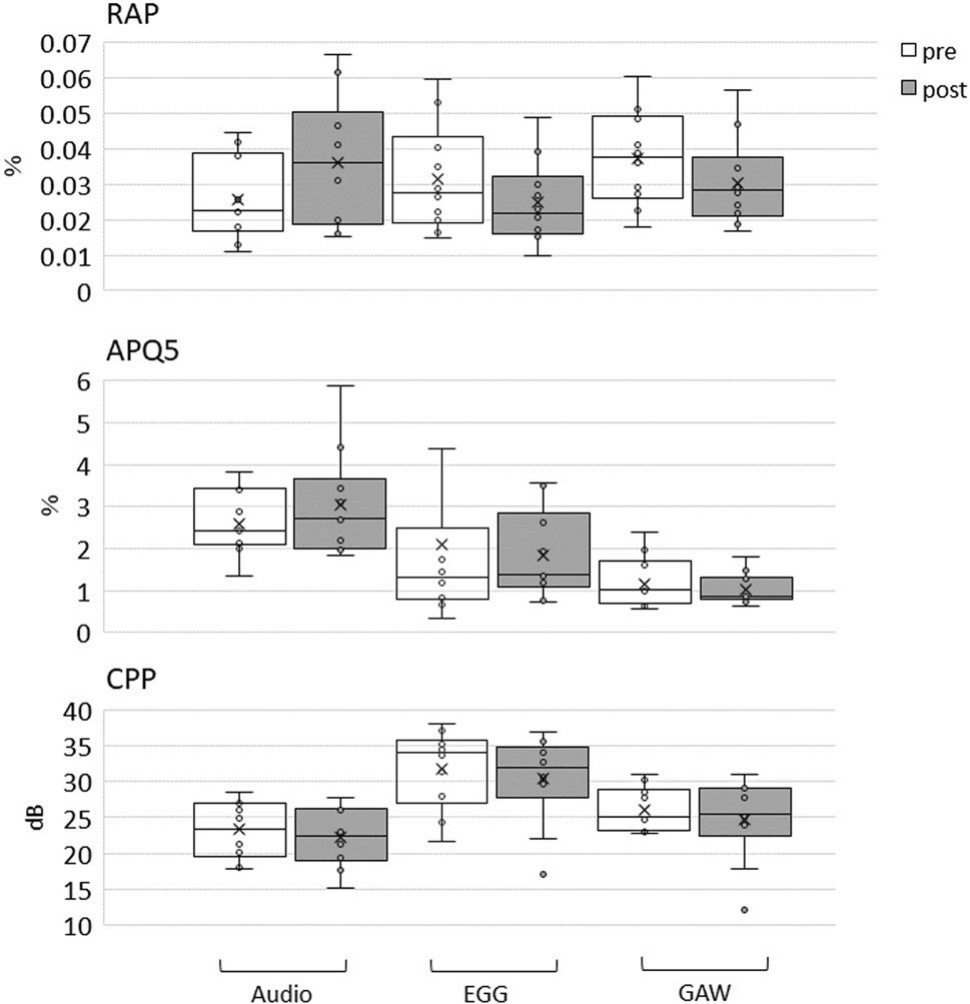
Audio, electroglottographic (EGG) and Glottal area waveform (GAW) derived Relative Average Perturbation (RAP), Amplitude Perturbation Quotient (APQ5) and Cepstral Peak Prominence (CPP) for pre and post intervention measurements

However, there were statistically divergent effects on perturbations measures. While RAP_GAW_ and RAP_EGG_ exhibited a statistically lower value after the intervention (RAP_GAW_ p value 0.05 and RAP_EGG_ with statistical tendency p value 0.1), RAP_Audio_ showed a statistically detectable rise after the intervention (p value 0.025). However, as shown in Fig. 6, the absolute difference was with mean values for RAP_GAW_ of 0.010%, RAP_EGG_ of – 0.007% and RAP_Audio_ of – 0.007% very small. Both, differences for APQ and CPP failed to reach statistically significance.

**Fig. 7:**
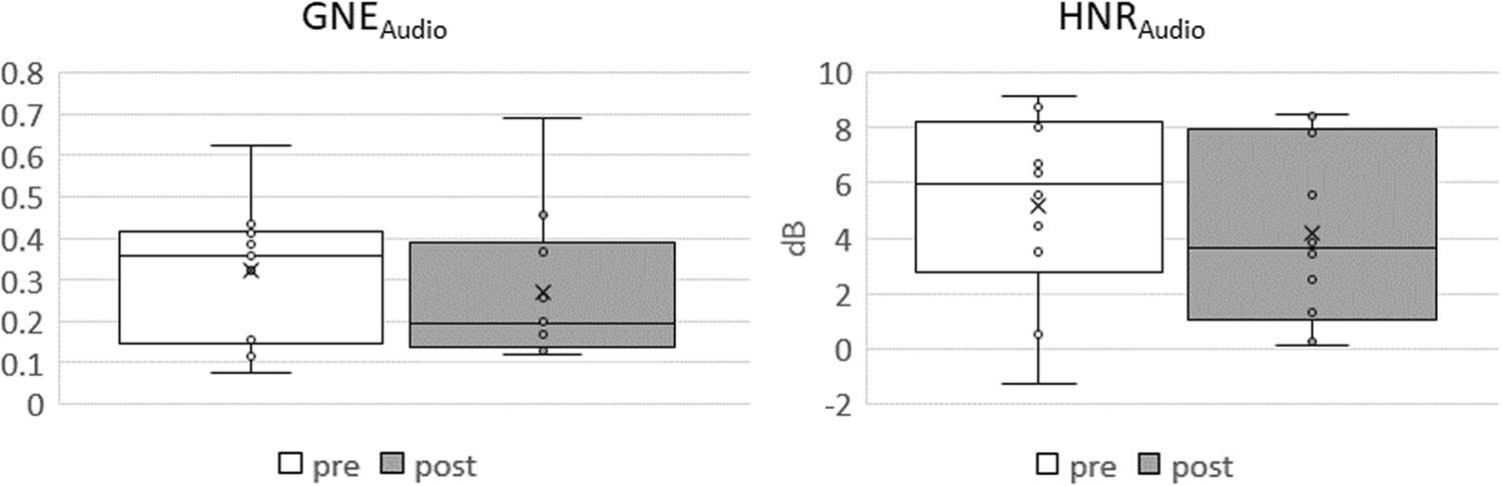
Audio derived Glottal to Noise Excitation Ratio (GNE) and Harmonic to Noise Ratio (HNR) for the pre and post intervention measurements

Furthermore, both values representing the signal to noise ratio, HNR_Audio_ and GNE_Audio_, were not found to be significantly different after the whisper loading test, Fig. 7.

## Discussion

This study explores the impact of a standardized whisper loading test on vocal fold oscillation characteristics and vocal function in vocally healthy subjects. Overall, only a few values exhibited statistically significant changes after the whispering intervention. Therefore, a substantial influence of whispering on vocal function seems rather unlikely, at least within the brief period of ten minutes whispering.

In contrast to voice production based on the myoelastic-aerodynamic principle, where air pulses are generated by the interruption of the transglottic air flow by the vocal folds, whispering follows an aero-acoustic principle. In this mechanism, turbulences occurring in the larynx produce vortices that generate noise, afterwards modulated by the resonances of the vocal tract. Vocal fold oscillations and the associated impact or shear stress on the vocal folds, are often absent during voiceless whispering [38]. However, whispering has been considered problematic to vocal function due to malregulations and misuse of control systems [41]. In this respect, it has been suggested that there is a difference between a relaxed and untensioned version of a whisper compared to a forced and tensioned version of a whisper. Based on this hypothesis, the presented study expected that if whispering influenced vocal function, this effect would be more pronounced in the group of the forced tensioned whisper. Consequently, a whisper loading test was designed analogous to a vocal loading test [8, 14]. Although the whisper loading test was tried out before the experiment in order to understand what could be considered as forced or tensioned, resulting in the 70 dB(A)@30 cm criterion, the presented data show that this criterion was not met by all subjects. In fact, only almost half of the subjects approached the criterion, while the other fell significantly lower regarding the 70 dB. To achieve such high dB levels during whispering, the authors expected that sound production should be related to tension, by means of increasing subglottic pressure, leading to greater flow and/or greater transglottic jet flow due to adduction. Therefore, this group was denoted as TW. However, it cannot be excluded that the UW group also had considerable tension, resulting in inefficiency concerning the whisper production. A limitation of the presented study is the absence of HSV verification during the whisper loading, which could have helped differentiate such noise production patterns.

The whisper loading test revealed no substantial differences in general vocal function, as measured by the DSI. However, a noticeable observation was the increase in minimum intensity after the intervention. The rise was with 2.4 dB almost in the same magnitude order as a rise of the minimum intensity after a 80 dB@30 cm vocal loading test for 10 min [32]. Because the minimum intensity could be related to the phonation threshold pressure [26, 27], it could be expected that greater tension in the vocal folds due to the loading might have produced this rise. In line with this, the TW group exhibited a rise of OQ_GAW_ and GGI for all subjects, potentially associated with either fatigue after the loading or as a residual effect of the whispering position in the larynx. However, there were no statistically significant changes in ClQ, SPL, HNR or GNE, i.e., not indicating a major decrease of vocal efficiency after the intervention. The difference of OQ_GAW_ and OQ_EGG_ was not totally unexpected. While the OQ_GAW_ is calculated from the laryngoscopic GAW from 2D images, OQ_EGG_ is derived from impedance changes of the 3D oscillating system, however also producing impedance changes when the glottis is not entirely closed. It has been shown before that there is a great agreement of OQ_EGG_ and OQ_GAW_ for OQ_GAW_ values up to 0.7 but a strong disagreement for values above, as measured in the presented study [49].

There were no major changes in periodicity in relation to the phonovibrograms. In fact, also some phase differences, i.e., subjects P1,3,4,5, and 10, cf. Figure 5, maintained such an anterior–posterior phase difference after the whisper intervention. However, RAP showed statistical differences after the intervention. While RAP_GAW_ and RAP_EGG_ were found lower after the intervention, suggesting a stabilizing effect, RAP_Audio_ was found to be increased. This difference was unexpected. It should be mentioned that all three signals are different: While the audio signal is modified by the resonatory properties of the vocal tract, the EGG is dependent on the electric impedance and thus the properties of the tissue which the current has to path, and the GAW is determined by the amount of pixels. All this modifies the signal amplitudes and configuration which has an effect on the estimation of the fundamental frequency and consecutively frequency perturbation. Still, it is essential to note that despite statistical significance the absolute differences were small.

The study raises the question of whether whispering might affect vocal function, particularly concerning post-phonosurgery. The presented data from vocally healthy subjects indicate that even for a forced and tensioned whisper, the effects lie within a negligible extent. However, it is crucial to acknowledge that the presented data only pertain to a single ten-minute loading interval. Also, it should be considered that whispering could result in vocal malregulations. Furthermore, it should be analyzed in future investigations if the data show differences for different types of whispering, i.e., relaxed or voiced. For such an experiment, many more subjects should be included because it could be expected that the effect size of this cohort is rather small. Lastly, it’s important to note that the presented data focuses on healthy subjects. Effects may differ in patients with vocal fold injuries. Consequently, we cannot fully conclude whether the different types of whispering are all harmless to patients with vocal fold injuries such as after phonosurgery. It appears therefore problematic to give recommendations concerning this group. However, it seems reasonable to believe that if whispering is allowed to patients, they should be introduced to an UW mechanism in order to avoid tissue stress.

### Limitations

There are some more limitations associated with the presented study. First, due to the complexity of the experimental setup, only a small number of subjects could be included. Also, as mentioned before, it is a limitation of this study that the whisper mechanisms haven’t been verified through HSV during the whisper loading, which could have helped differentiate the noise production patterns. This prevented a validation of TW in contrast to UWFurthermore, there was no follow up analysis conducted several minutes after the whisper loading task. It could be relevant to investigate whether the observed effects disappear after a short time interval. In the presented study, efforts were made to normalize *ƒ*_o_. Although not statistically significant, not all participants achieved the expected *ƒ*_o_. It could be a subject for further research to analyze possible changes in *ƒ*_o_ when it is not normalized but freely chosen.

## Abbreviations

APQ: Amplitude Perturbation Quotient
ClQ: Closing Quotient
CPP: Cepstral Peak Prominence
dB: Decibel
DSI: Dysphonia Severity Index
EGG: Electroglottography
*f*_*o*_: Fundamental Frequency
GAW: Glottal Area Waveform
GGI: Glottal Gap Index
GNE: Glottal to Noise Excitation Ratio
HNR: Harmonics to Noise Ratio
OQ: Open Quotient
PVG: Phonovibrogram
RAP: Relative Average Perturbation
SPL: Sound Pressure Level
HSV: High Speed Videolaryngoscopy
TW: Tensioned Whisper
UW: Untensioned Whisper
VHI: Voice Handicap Index
